# Supplementary material for: Transcriptional control of a stem cell factor nucleostemin in liver regeneration and aging
Source: PLoS One. 2024 Sep 11;19(9):e0310219. doi: 10.1371/journal.pone.0310219 (PMC11389944; doi:10.1371/journal.pone.0310219)
Supplement: S1 Fig — (PDF) [file pone.0310219.s001.pdf]

Figure S1

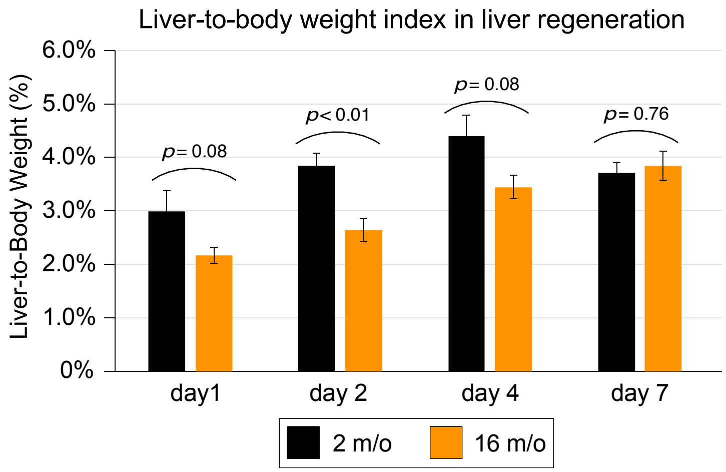

Liver-to-body weight index in liver regeneration
Liver-to-Body Weight (%)
6.0%
5.0%
4.0%
3.0%
2.0%
1.0%
0%
p= 0.08
p< 0.01
p= 0.08
p= 0.76
day1
day 2
day 4
day 7
2 m/o
16 m/o

Figure S1. Liver-to-body weight indices during liver regeneration at 2 and 16 months old. Liver-to-body weight ratios during the regeneration of mouse livers at 1, 2, 4, and 7 days after receiving 70% partial hepatectomy at 2 (black bars) and 16 (orange bars) months old. Bars show mean ± s.e.m. with p values indicated on top.
