# Supplementary material for: Transcriptional control of a stem cell factor nucleostemin in liver regeneration and aging
Source: PLoS One. 2024 Sep 11;19(9):e0310219. doi: 10.1371/journal.pone.0310219 (PMC11389944; doi:10.1371/journal.pone.0310219)
Supplement: S2 Fig — (PDF) [file pone.0310219.s002.pdf]

Figure S2

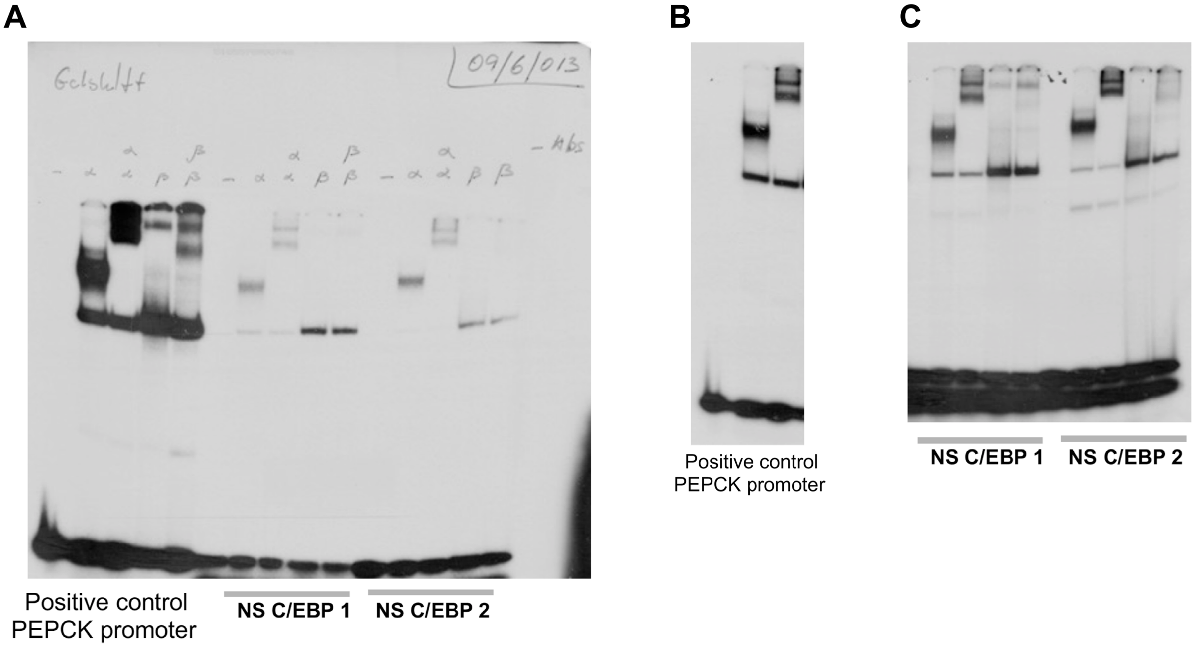
A
09/6/013
Positive control
PEPCK promoter
NS C/EBP 1
NS C/EBP 2
B
Positive control
PEPCK promoter
C
NS C/EBP 1
NS C/EBP 2

Figure S2. Uncropped and unadjusted images of EMSA data shown in Figure 4A.

(**A**) Uncropped and unadjusted EMSA data of C/EBPA binding to the PEPCK promoter and NS promoter probes. (**B**) Short exposure of EMSA data of C/EBPA binding to a PEPCK promoter probe (positive control). (**C**) Long exposure of EMSA data of C/EBPA binding to the NS-S1 and NS-S2 probe.
