# Supplementary material for: Transcriptional control of a stem cell factor nucleostemin in liver regeneration and aging
Source: PLoS One. 2024 Sep 11;19(9):e0310219. doi: 10.1371/journal.pone.0310219 (PMC11389944; doi:10.1371/journal.pone.0310219)
Supplement: S3 Fig — (PDF) [file pone.0310219.s003.pdf]

Figure S3

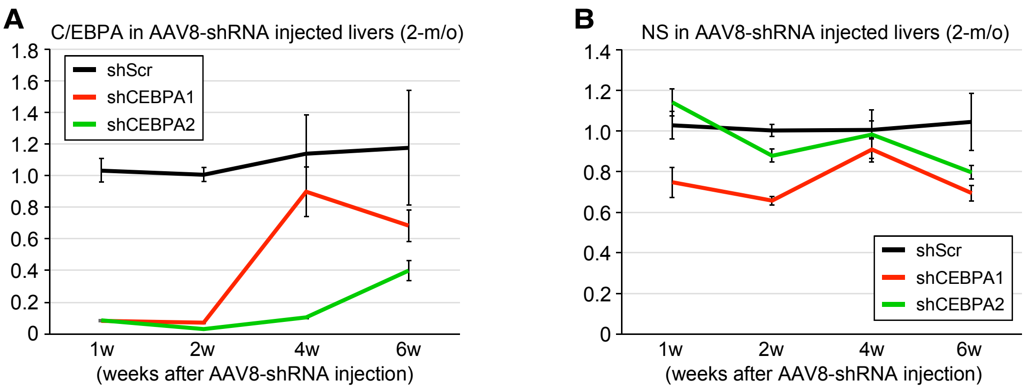

A
C/EBPA in AAV8-shRNA injected livers (2-m/o)
1.8
1.6
1.4
1.2
1.0
0.8
0.6
0.4
0.2
0
shScr
shCEBPA1
shCEBPA2
1w
2w
4w
6w
(weeks after AAV8-shRNA injection)
B
NS in AAV8-shRNA injected livers (2-m/o)
1.4
1.2
1.0
0.8
0.6
0.4
0.2
0
shScr
shCEBPA1
shCEBPA2
1w
2w
4w
6w
(weeks after AAV8-shRNA injection)

Figure S3. Hepatic expression of C/EBPα and NS in 2-m/o mice injected with AAV8-shRNA. Line graphs of the results shown in Fig. 6B1 and Fig. 6C1, showing hepatic expression of C/EBPα (**A**) and NS (**B**) in 2-m/o mice injected with AAV8-shScr (black line), AAV8-shCEBPA1 (red line), or AAV8-shCEBPA2 (green line) and sacrificed at 1 week (1w), 2w, 4w, or 6w after AAV8 injection.
